# Development and validation of the nursing confidence in managing sedation complications scale

**DOI:** 10.1002/nop2.725

**Published:** 2021-01-28

**Authors:** Aaron Conway, Kristina Chang, Navpreet Kamboj, Joanna Sutherland

**Affiliations:** ^1^ Peter Munk Cardiac Centre University Health Network Toronto ON Canada; ^2^ Lawrence S. Bloomberg Faculty of Nursing University of Toronto Toronto ON Canada; ^3^ School of Nursing Queensland University of Technology (QUT) Brisbane Qld Australia; ^4^ Rural Clinical School University of New South Wales Coffs Harbour NSW Australia; ^5^ Department of Anaesthesia Coffs Harbour Health Campus Coffs Harbour NSW Australia

**Keywords:** conscious sedation, deep sedation, item response theory, nursing, patient safety, scale development, self‐efficacy

## Abstract

**Aim:**

To develop the Nursing Confidence in Managing Sedation Complications Scale.

**Design:**

A multi‐phased approach was used.

**Methods:**

An initial bank of items was created based on the authors' experience and clinical practice guidelines. An expert panel assessed content validity. Exploratory factor analysis was used for item reduction and regression was used to explore construct validity. Responsiveness was evaluated using a pre‐test post‐test design.

**Results:**

Criteria for content validity was met for 34 items. An 18‐item, three‐factor solution was identified from exploratory factor analysis performed using Nursing Confidence in Managing Sedation Complications Scale scores from 228 nurses. Subscales accounted for 66% of the variance. Cronbach's alpha for the scale (0.95) and subscales was high (>0.85). There were differences (*p* < .001) in Nursing Confidence in Managing Sedation Complications Scale scores relative to years of experience and work environment. NC‐MSCS scores increased significantly from before to after sedation training (mean difference = 31.8; 95% CI = 24.4–39; *N* = 31).

## 1 INTRODUCTION

Sedative and analgesic medications can be administered to manage pain, discomfort and anxiety or distress during diagnostic and/or interventional procedures that do not require general anaesthesia. This practice is referred to as procedural sedation and analgesia (PSA) (Apfelbaum et al., 2018). It is common for nurses to administer the sedative and analgesic medications according to prescription from the medical practitioner performing the procedure and to monitor the patient (termed nurse‐administered PSA) (Conway et al., 2014). Complications are known to arise during PSA due to side effects of sedative and analgesic medications (Apfelbaum et al., 2018). For example, the sedative medications can cause relaxation of muscles and hence loss of ability to maintain a patent airway. The analgesic medications used (typically the opioids) can also cause the breathing rate to be depressed. If such complications are not detected and corrective interventions applied, the level of oxygen circulating in the blood can drop to a level that is insufficient for the body's requirements (a condition known as hypoxaemia). If severe enough and left uncorrected, hypoxaemia can result in permanent damage to vital organs, such as the brain and heart, potentially causing serious adverse events, such as permanent neurological disability or death. Intervening at or soon after the onset of complications can prevent these adverse events from occurring. Therefore, prompt detection and treatment of sedation‐related complications is vital to ensure patient safety during procedures performed with PSA (Bhananker et al., 2006).

## 2 BACKGROUND

Self‐efficacy is a measure of one's beliefs in their own capability to produce given attainments (Bandura, 1995). Previous research has demonstrated that people will exert maximal effort and persist despite failure if they believe they are capable in completing a given task (Bandura et al., 1999). In the healthcare context, it has been shown that clinicians who lack confidence in their abilities do not take needed actions for their patients (Johnson & Kurtz, 2001). Therefore, it is possible that nurses may not take actions to manage sedation‐related complications if they lack confidence in their abilities to complete the necessary tasks. Despite clear relevance, there has been no research about nurses' self‐efficacy in managing the complications that arise due to side effects of sedation and analgesic medications administered during medical procedures. For this reason, the aim of this study is to develop an instrument that measures nurses' self‐efficacy in managing complications that are known to arise due to side effects of sedative and analgesic medications administered during medical procedures. We have named this instrument the Nursing Confidence in Managing Sedation Complications Scale (NC‐MSCS). Once validated, the NC‐MSCS could be used in practice to inform the development and evaluation of nursing education programs about the management of complications during sedation. The scale could also be used in the research context as an instrument to compare the effectiveness of different nursing education programs about procedural sedation in improving nursing confidence.

## 3 METHODS

The NC‐MSCS was developed and validated through three phases. Content validity of the items was evaluated in the first phase with input from an expert panel of clinicians with expertise in procedural sedation. In the second phase, a survey study was undertaken for item reduction and to evaluate the structural validity of the scale. In the third phase, responsiveness was evaluated using a pre‐test post‐test design. The STROBE checklist was used to guide the reporting of results.

### 3.1 Participants

#### 3.1.1 Expert panel

Clinicians who were known to the authors as having expertise in the area of nurse‐administered sedation were invited to participate. These included senior clinicians who have developed education programs or competency assessments for the administration of procedural sedation and analgesia or researchers who have published on the topic.

#### 3.1.2 Online survey

Inclusion criteria for this phase of the study were nurses who administer, or monitor patients who have received, procedural sedation and analgesia during a medical procedure. An invitation to participate with a link to an online survey was distributed through Interventional Nursing Council of the Cardiac Society of Australia and New Zealand, Australian College of Operating Room Nurses, Medical Imaging Nurses Association, Gastroenterological Nurses College of Australia, Thoracic Society of Australia and New Zealand Respiratory Nurses Special Interest Group and the College of Emergency Nursing Australia.

#### 3.1.3 Pre‐test post‐test study

Nurses who were undergoing a one‐day procedural sedation education session delivered by one of the authors (JS) were invited to participate. The course involved pre‐reading, based on previously described Minimum Standards for Safe Procedural Sedation (https://www.aci.health.nsw.gov.au/resources/anaesthesia‐perioperative‐care/sedation/safe‐sedation‐resources). The face‐to‐face teaching included practical examples for risk assessment, airway management, team communication and behaviours including graded assertiveness and crisis management and escalation.

### 3.2 Measurement tools

#### 3.2.1 Expert panel

An initial bank of items for potential inclusion in the NC‐MSCS was created. Items were chosen based on the authors' clinical experience as well as from clinical practice guidelines for procedural sedation (Apfelbaum et al., 2018; Conway et al., 2013a, 2013b, 2014; Sutherland et al., 2020). Each item was prefixed with the phrase: “I am confident I am able to…”. Responses were measured on a 7‐point scale, ranging from “strongly disagree” to “strongly agree.”

The expert panel assessed the initial bank of NC‐MSCS items for relevance using a 4‐point scale (1 = not relevant; 2 = somewhat relevant; 3 = quite relevant; 4 = highly relevant). In addition, there was free‐text space for the expert panel participants to provide suggestions to increase clarity of the wording of the items and to suggest additional items. The draft bank of items comprised four conceptual domains with a total of 37 items. Domain one included 6 items that measure nurses' confidence in identifying patients who are at greater risk of a sedation‐related complication based on a pre‐procedural assessment. Domain two included 14 items that measure nurses' confidence in identifying and responding to sedation‐related complications. Domain three included 11 items that measure nurses' confidence in identify the situations where different interventions to treat sedation‐related complications may be required. Domain four included 7 items that measures nurses' confidence in the technical skills required for interventions to treat sedation‐related complications.

#### 3.2.2 Online survey

##### NC‐MSCS

The items identified by the expert panel as being relevant to the measurement of nursing confidence in managing the complications of sedation were included in the survey. They were presented to respondents participating in the online survey in a random order.

##### Sedation experience and education

Nurses were asked a series of questions to ascertain the extent of the experience and education in aspects related to the management of sedation‐related complications.

##### General Self‐efficacy

The New General Self‐efficacy scale (NGSE) was also included in the online survey (Chen et al., 2001). It consists of eight items that has demonstrated high reliability, discriminant validity and predictive validity (Chen et al., 2001). The NGSE scale is scored on a 5‐point scale from strongly disagree (1)–strongly agree (5).

#### 3.2.3 Pre‐test post‐test study

Time was allocated at the start and end of the education session to allow those nurses who chose to take part in the study to complete the NC‐MSCS. In addition to the NC‐MSCS, participants responded to a set of items designed to ascertain the extent of their experience and education in aspects related to the management of sedation‐related complications. Participants were also asked to rate the extent to which their overall knowledge and confidence changed from before to after the sedation training, using a seven‐point rating scale from greatly decreased (1)–greatly increased (7).

### 3.3 Statistical considerations and data analysis

#### 3.3.1 Sample size considerations

We aimed to recruit 8–10 participants for the expert panel survey, as has been recommended for content validation (Polit et al., 2007). We aimed to recruit 200 participants for the nurse survey. A sample of this size met a recommended minimum necessary sample size for factor analysis (assuming the variables‐to‐factors ratio was not lower than 5) (Mundfrom et al., 2005). For the pre‐test post‐test phase of the study, the total number of participants was limited by the number of nurses participating in the education sessions. Therefore, no specific power analysis was performed to calculate the sample size.

#### 3.3.2 Statistical methods

All analyses were conducted using R (R Core Team, 2020). For the expert panel survey, ratings of the relevance of items were used to compute the content validity index for each item by averaging the proportion of respondents that rated the item as a 3 or 4 (i.e. “quite relevant” or “highly relevant”) (Polit et al., 2007). Items with a content validity index of <0.78 were considered for revision and those with very low index values were considered for deletion (Polit et al., 2007). Scale‐level content validity index was also calculated by averaging across items (Polit & Beck, 2006). In addition, the research team revised the wording of items based on panel members' responses regarding the clarity of items.

For item reduction and examination of the structural validation of the NC‐MSCS, the following analyses were undertaken using nurses' responses from the online survey. Ordinary least squares estimations with a matrix of polychoric correlations were generated for an exploratory factor analysis due to the use of ordinal response variables (Gaskin & Happell, 2014). Parallel analysis was used to determine the optimal number of factors for extraction and principal axis factoring with promax rotation was used for factor extraction. Internal consistency, calculated using Cronbach's alpha coefficient, was calculated to examine the reliability of the scale. There was <2% missing data for all scale items. We imputed missing values using expectation maximization (Fox‐Wasylyshyn & El‐Masri, 2005).

Construct validity was evaluated by using multivariable linear regression to identify associations between scores on the NC‐MSCS and nurses' experience and education regarding sedation. We hypothesized that higher scores on the NC‐MSCS would be associated with:

- nurses who reported more years of experience administering sedation
- nurses who reported more years of experience in nursing
- nurses who worked in departments that provided formal education in sedation
- nurses who were certified in ALS
- nurses who administered sedation more frequently
- nurses who have higher ratings for general self‐efficacy (measured using the NGSE) (Chen et al., 2001)
- nurses who rated their knowledge about sedation higher
- nurses who worked in critical care (emergency department/ICU/PACU) or anaesthesia.

To evaluate responsiveness, analyses focused on testing the hypothesis that there would be a positive correlation between the change in participants NC‐MSCS scores and changes in participant ratings of their overall knowledge and confidence in managing sedation complications from before to after undertaking a training course in sedation. Linear regression was used to determine the association between the change in NC‐MSCS scores from pre‐ to post‐training and the extent to which participants' perceived their overall knowledge and confidence in managing PSA complications had changed. We used a Tufte Slopegraph to visually display the change in NC‐MSCS scores from the pre‐ to post‐time‐points and used bootstrap estimation (5,000 resamples) to calculate the mean difference with 95% confidence intervals (Ho et al., 2019). A standardized response mean (mean change divided by the standard deviation of the change score) was also calculated.

### 3.4 Ethics

Ethical approval was provided by the Queensland University of Technology Human Research Ethics Committee (61986 & 56161).

## 4 RESULTS

### 4.1 Content validity

We received responses from nine participants for the expert panel. Pre‐specified criteria for content validity were met with the item‐content validity index being higher than 0.78 for 34 items and the scale content validity index was 0.91.

### 4.2 Structural validity

Usable data for the analyses were obtained from 228 nurses. Demographic characteristics are presented in Table 1. Most of the sample was highly experienced both in terms of total years in nursing and the number of years of experience with procedural sedation.

**TABLE 1:** Participant characteristics for online survey

Characteristic	*N* = 228^a^
What is your specialty area of practice?	
CCL	28 (12%)
Radiology	27 (12%)
Endoscopy	72 (32%)
Bronchoscopy	2 (0.9%)
Surgery	20 (8.8%)
Office‐based surgery	2 (0.9%)
Emergency	9 (4.0%)
Anaesthesia/Recovery	27 (12%)
Other	3 (1.3%)
ICU	37 (16%)
Unknown	1
Years of experience in nursing	28 (16, 35)
Unknown	34
Are you currently certified in Advanced Life Support? (i.e. completed a course within the last 12 months)	139 (61%)
Unknown	1
Which type of hospital do you work in?	
Private	81 (36%)
Public	144 (64%)
Unknown	3
Years of experience with procedural sedation	14 (8, 20)
Unknown	44
How frequently do you either administer, or monitor patients who have received, procedural sedation and analgesia (conscious sedation) in a typical working week?	
Never	6 (2.6%)
Rarely	13 (5.7%)
Sometimes	38 (17%)
Often	106 (47%)
Always	64 (28%)
Unknown	1
Received education about sedation	88 (39%)
Unit/hospital has a policy about sedation	142 (62%)
Required to undergo competency assessment in sedation	49 (21%)
Rate your overall knowledge in identifying and treating sedation‐related complications (0 = ‘No knowledge’ to 100 = ‘Very knowledgable’)	80 (70, 90)
Unknown	17
Rate your overall degree of confidence in identifying and treating sedation‐related complications (0 = ‘No confidence’ to 100 = ‘Very confident’)	80 (70, 90)
Unknown	16

^a^ Statistics presented: *N* (%); median (IQR).

**FIGURE 1:**
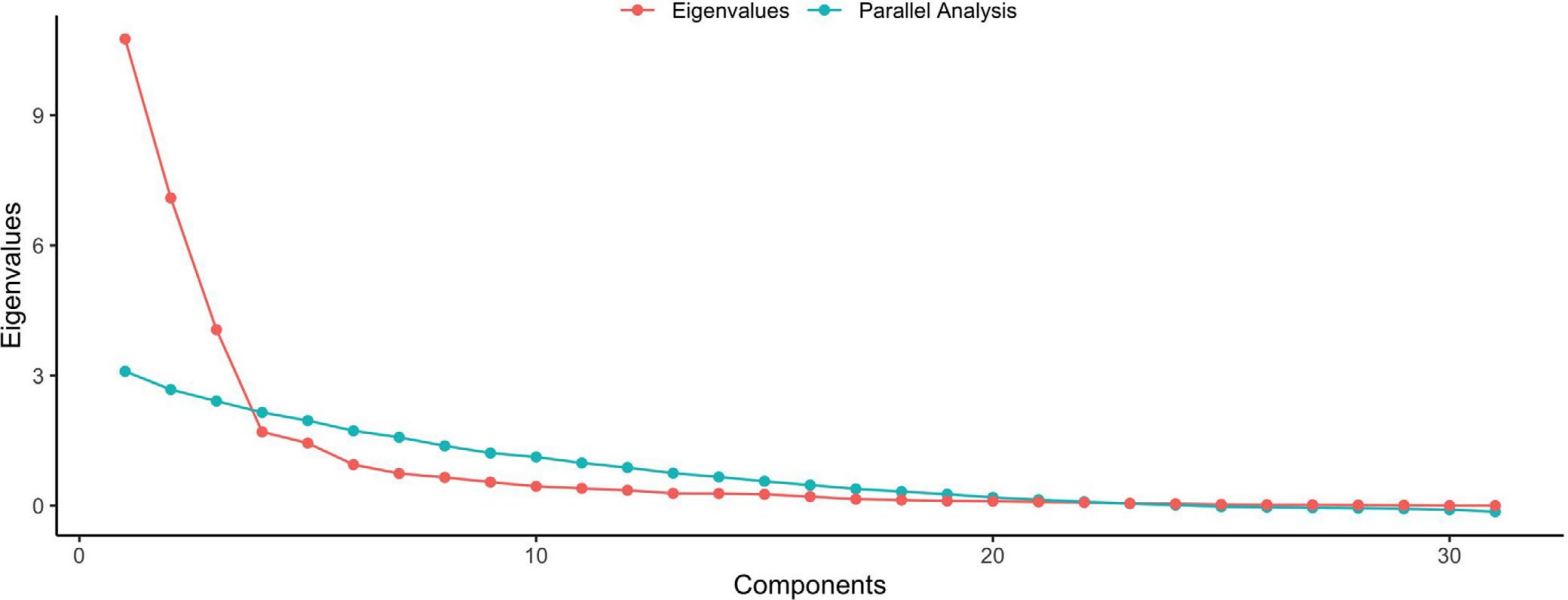
Scree plot from initial factor analysis of the Nursing‐Confidence in Managing Sedation Complications Scale

**FIGURE 2:**
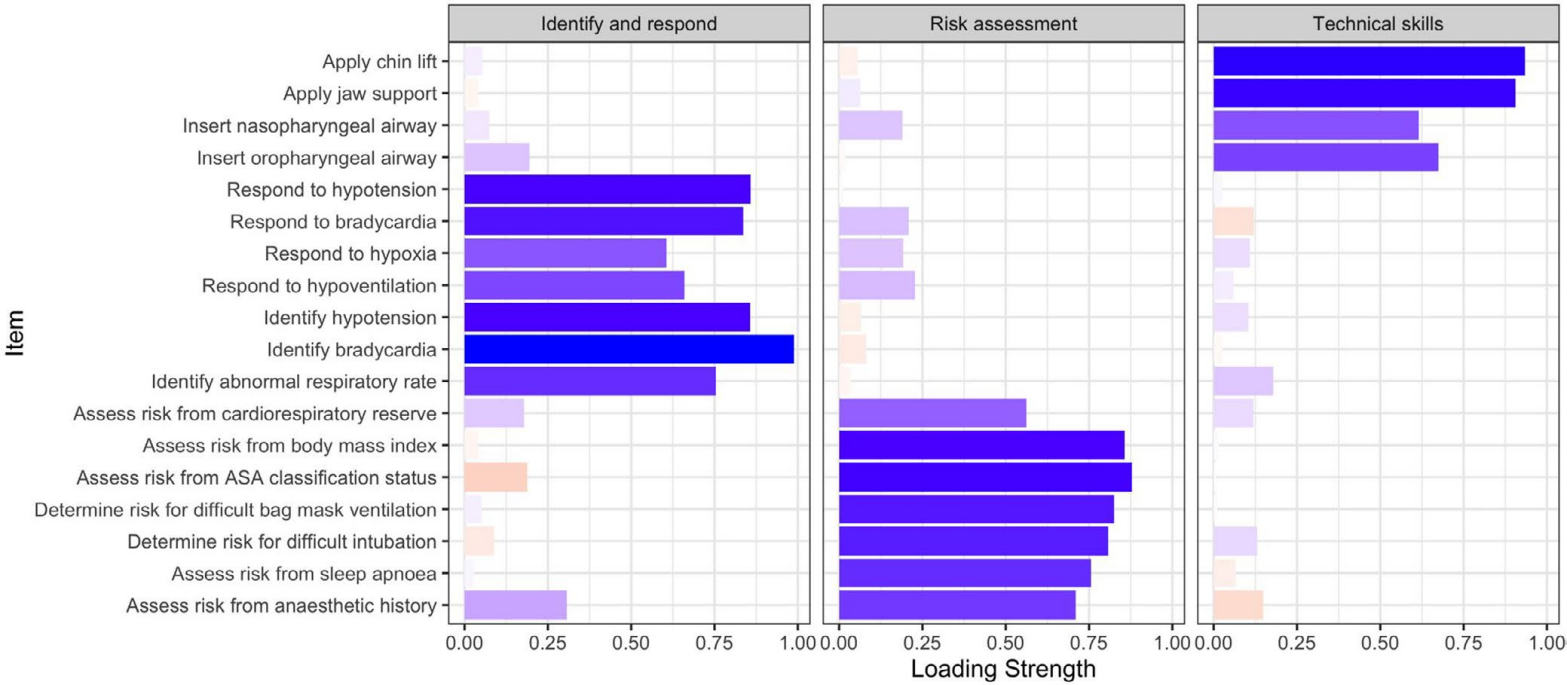
Rotated factor loadings for the Nursing Confidence in Managing Sedation Complications Scale

**FIGURE 3:**
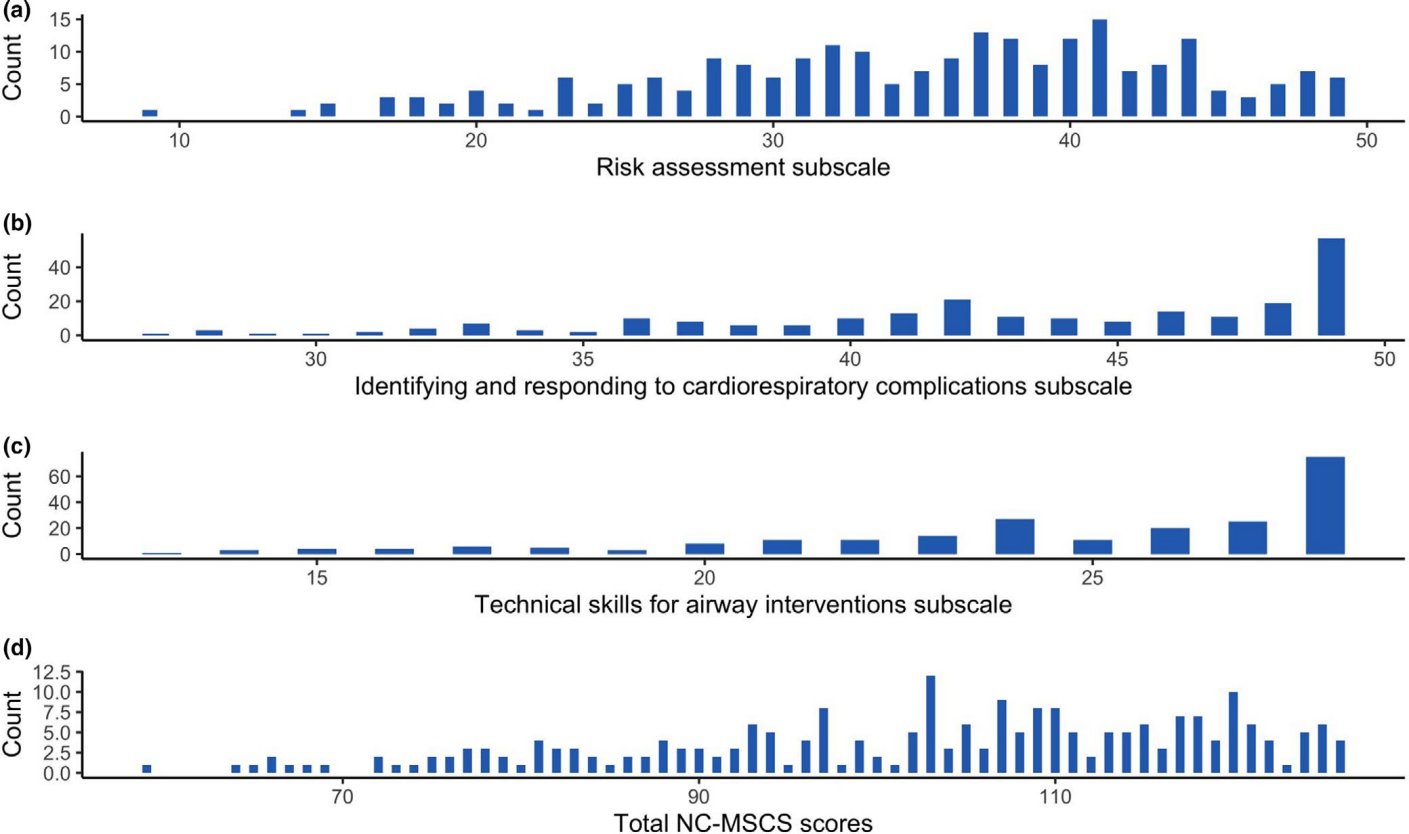
Distribution of Nursing Confidence in Managing Sedation Complications Scale and subscale scores in the online survey

Before the main analysis, the appropriateness of factor analysis was considered. The Kaiser‐Meyer‐Olkin measure of sampling adequacy was high at 0.95. Examination of the scree plot (Figure 1) and parallel analysis indicated that a 3‐factor solution was appropriate. Items that did not meet criteria were deleted one at a time with factor analysis repeated after each item deletion. We repeated this process until all items retained had a factor loading of at least 0.40 and loaded on one coherent factor. The original scale was reduced from 34–18 items (Figure 2). Eight items were removed because there were high loadings (>0.35) on more than one subscale, with loading differences <0.15. A further eight items were removed because the items did not load on a conceptually consistent subscale. For example, the item “I am confident I am able to identify laryngospasm,” loaded with items related to risk assessment. Subscales were named (1) Risk assessment; (2) Identifying and responding to cardiorespiratory complications; and (3) Technical airway skills. The subscales together accounted for 65.6% of the variance in the scale. Cronbach's alpha for the 18‐item scale (Cronbach's *α* 0.95) and each subscale was high (Cronbach's *α* > 0.85). The minimum score for the 18‐item NC‐MSCS is 18 and maximum is 126. The distribution of scores is presented in Figure 3. The technical airway skills and identifying and responding to cardiorespiratory complications subscales were heavily right‐skewed.

### 4.3 Construct validity

Construct validity was established with significant differences (*p* < .001) in NC‐MSCS scores relative to years of nursing experience and specialty area of practice (critical care/anaesthesia nurses reported higher NC‐MSCS scores than procedural/operating theatre nurses) (Table 2). Statistically significant positive correlations between NC‐MSCS scores and participant ratings of knowledge and confidence in managing procedural sedation as well as perceived general self‐efficacy were also observed (Figure 1).

**TABLE 2:** Multivariable regression model of associations between NC‐MSCS score and participant characteristics

Variable	*β*	*p*‐value	Lower 95% CI	Upper 95% CI
(Intercept)	88.749	.000	69.220	108.278
Years in nursing	0.376	.000	0.179	0.574
Years of experience administering sedation	0.027	.846	−0.245	0.299
Frequency of administering sedation in a typical working week	0.035	.974	−2.081	2.151
Trained in Advanced Life Support	−2.588	.207	−6.626	1.450
Received education about sedation	1.403	.483	−2.543	5.348
New General Self‐efficacy Score	−1.181	.000	−1.704	−0.658
Works in a critical care environment	8.792	.000	4.630	12.953
Perceived knowledge about managing sedation‐related complications	0.262	.000	0.138	0.386

### 4.4 Responsiveness

**FIGURE 4:**
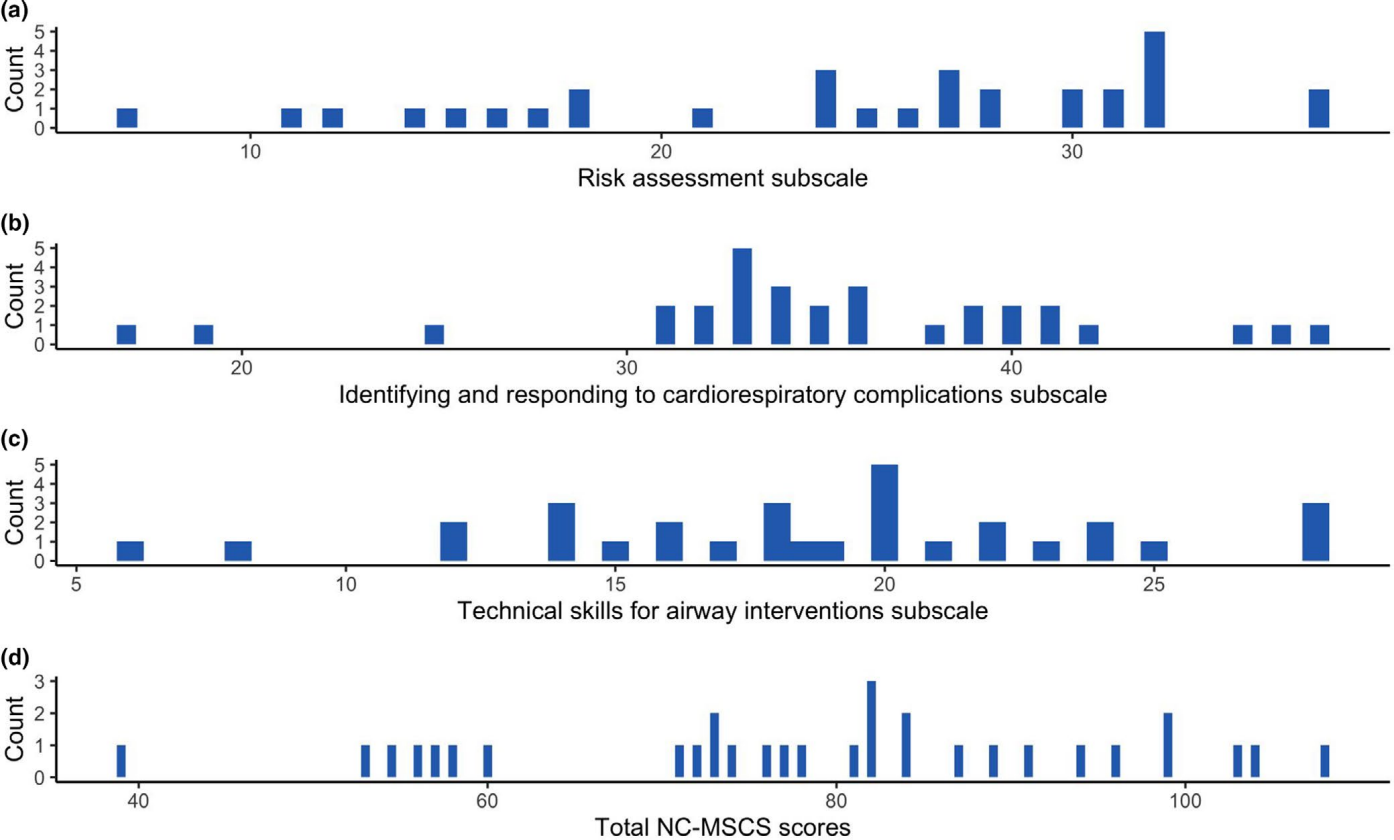
Pre‐sedation training distribution of Nursing Confidence in Managing Sedation Complications Scale and subscale scores

**FIGURE 5:**
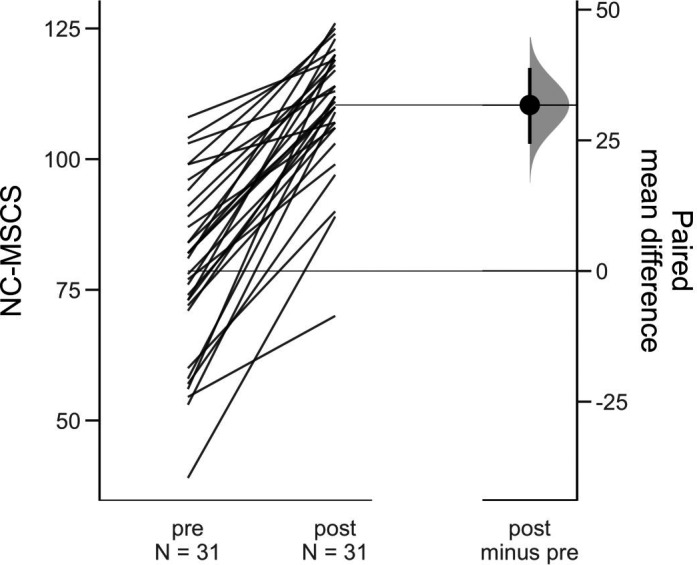
Change in Nursing Confidence in Managing Sedation Complications Scale scores from pre‐ to post‐training

**FIGURE 6:**
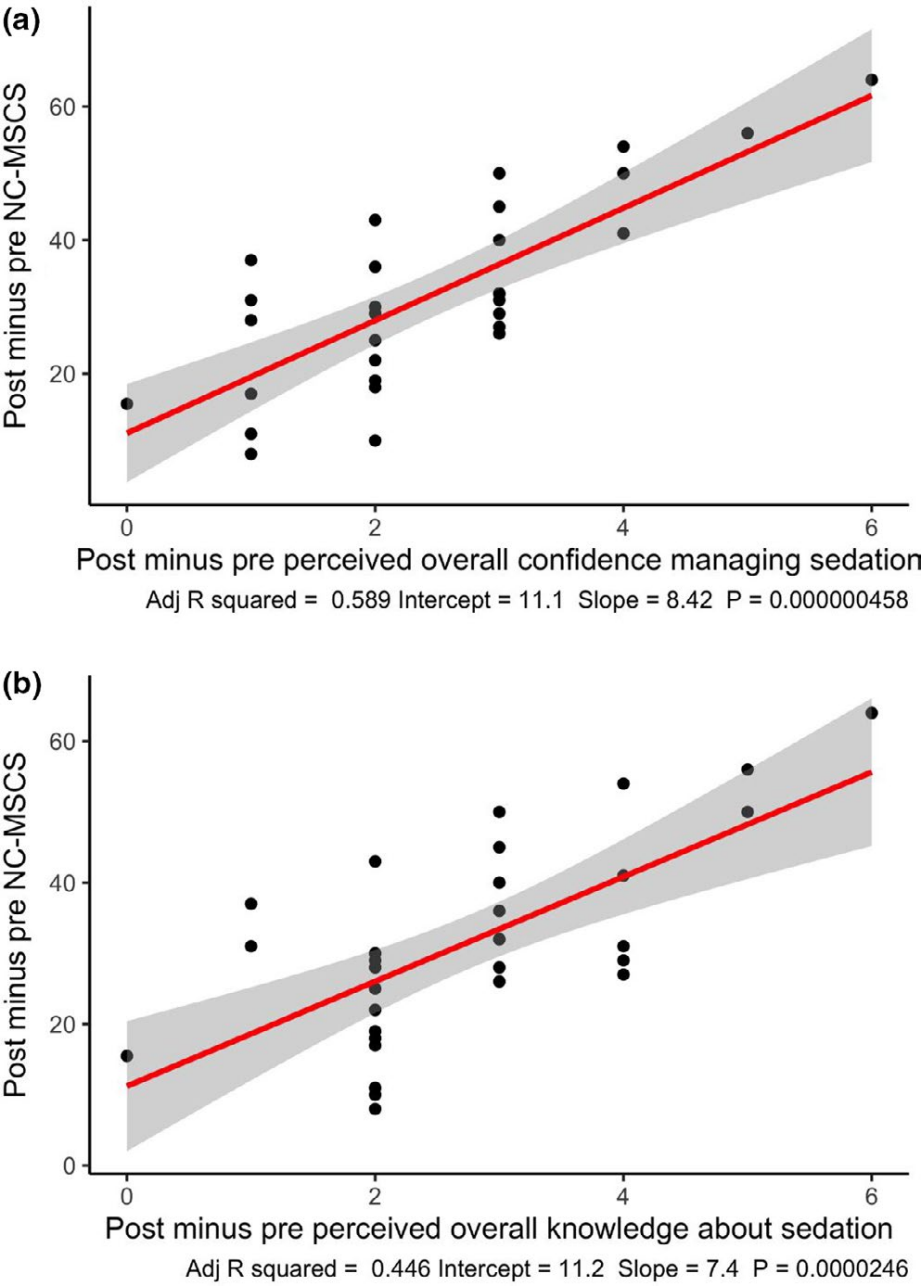
Association between change in Managing Sedation Complications Scale scores from pre‐ to post‐training with (a) change in overall rating of confidence in managing sedation; and (b) change in overall knowledge about sedation

A total of 31 nurses participated in the pre‐test post‐test study. Characteristics for participants included in this phase of the study are displayed in Table 3. On average, participants had approximately 4 years of experience administering sedation but reported only infrequently being required to administer or monitor patients who have received procedural sedation in a typical working week. Score distributions for each subscale and the total scale for the pre‐test are presented in Figure 4. The change in NC‐MSCS scores, displayed in Figure 5, increased significantly from pre‐ to post‐training in sedation (mean difference = 31.8; 95% CI = 24.4–39). In addition, the change in NC‐MSCS scores was positively correlated with the change in participants' ratings of overall confidence and knowledge about management of PSA complications (Figure 6). The standardized response mean, adjusted for the correlation between pre‐ and postmeasurements, was 1.3.

**TABLE 3:** Participant characteristics for pre‐test post‐test study

Characteristic	*N* = 31^a^
Specialty	
Anaesthesia/Recovery	3 (9.7%)
CCL	12 (39%)
Emergency	1 (3.2%)
Endoscopy	1 (3.2%)
ICU	5 (16%)
Medical ward	6 (19%)
Surgery	3 (9.7%)
Years of experience in nursing	6 (4, 12)
Unknown	1
Years of experience with procedural sedation	4 (1, 6)
Unknown	2
How frequently do you either administer, or monitor patients who have received, procedural sedation and analgesia (conscious sedation) in a typical working week?	
Always	5 (16%)
Never	2 (6.5%)
Often	4 (13%)
Rarely	3 (9.7%)
Sometimes	17 (55%)
Trained in advanced life support	13 (42%)

^a^ Statistics presented: *N* (%); median (IQR).

## 5 DISCUSSION

The aim of this study was to develop a scale to measure nursing confidence in managing sedation‐related complications. A large sample of nurses with diverse clinical experience across the numerous specialties where sedation is frequently used participated in the study. This provided the opportunity to eliminate redundancy from an initial comprehensive item pool, which was created by the authors of this study but refined by an expert panel using a rigorous process for establishing content validity (Polit et al., 2007). The resultant 18‐item NC‐MSCS demonstrated a robust 3‐factor structure with high internal consistency overall, as well as for each subscale measuring the domains of confidence in risk assessment, identification and responses to cardiorespiratory complications and application of airway interventions. Importantly, the scale was able to distinguish between‐group differences based on specialty expertise. Nurses who worked in critical care areas (defined as ICU, emergency or anaesthesia/recovery) reported considerably higher levels of confidence in managing sedation‐related complications compared with nurses who worked in other specialties (e.g. cardiac cath laboratory, radiology, endoscopy surgery). Effect sizes for associations between NC‐MSCS and years of experience in nursing, general self‐efficacy and general knowledge about managing sedation‐related complications were statistically significant but very small.

The right‐skewed distribution of subscale scores in the online survey should be noted. We used polychoric correlations for the exploratory factor analysis, which is the recommended approach for skewed data (Eijk & Rose, 2015; Gaskin & Happell, 2014). Although the potential for a ceiling effect should not be ruled out, it is reassuring that the distribution of scores in the pre‐sedation training group was not right‐skewed. It is likely the higher ratings for items within these particular subscales that were observed in the online survey were associated with specific characteristics of the sample, such as participants' years of experience in nursing and the clinical settings where the participants practiced (e.g. critical care setting versus procedural/medical settings).

The sample for the online survey was highly experienced and reported high levels of confidence in managing sedation complications. A convenience sampling approach and recruitment of participants through professional nursing organizations is the likely cause. The sample is therefore not entirely representative of the broader population of nurses who are involved in the management of procedural sedation. Prior research has identified that although sampling through professional organizations is a convenient approach to access a more broadly geographically distributed sample, it does not adequately represent the characteristics of the population (Gillespie et al., 2010). Conducting confirmatory factor analysis with a different sample of nurses would be useful to determine if the factor structure we identified is robust to sampling procedures.

If adjusted for the correlation between pre‐ and post‐measurements, Cohen's rule‐of‐thumb thresholds for interpreting effect size can be applied for the standardized response mean, which was one statistic we used to assess responsiveness of the NC‐MSCS (Cohen, 2013; Sivan, 2009). Results indicate there was a large improvement in confidence in the pre‐test post‐test study (SRMadj 1.3). In addition, there was a positive and strong relationship between changes in the NC‐MSCS from pre‐ to post‐training with perceived changes in overall knowledge and confidence. It is reassuring that the NC‐MSCS was able to detect a change in participants' confidence after undertaking a formal education session in management of sedation. Undertaking further investigations, such as test‐retest reliability to determine the minimal detectable difference in NC‐MSCS scores, would be required in order for this scale to be used with confidence as a measure to judge the effectiveness of particular sedation training programs/modes of sedation training. For example, considering the not insignificant costs associated with in‐person training, there may be advantages to delivering all or part of sedation education using more affordable and accessible online virtual modalities (Tobin et al., 2013). One way the efficacy of these alternative approaches could be compared would be to use the NC‐MSCS as an outcome measure.

### 5.1 Limitations

Respondents in the online survey were relatively homogenous in regard to having a large amount of experience in sedation as well as being highly experienced nurses. We recommend future testing of the NC‐MSCS in samples with greater heterogeneity. A convenience sampling approach was used for the pre‐test post‐test study. As such, selection bias may have influenced the results of the analyses conducted to evaluate the responsiveness of the NC‐MSCS. A small number of participants in the online survey reported that they would not administer or monitor patients who have received procedural sedation in a typical working week. We chose not to exclude these patients from the analyses because it is possible that they did have experience using procedural sedation, but just do not currently use it during a typical working week. Further psychometric testing to establish criterion‐related validity and test‐retest reliability would strengthen confidence in the usefulness of the NC‐MSCS.

## 6 CONCLUSION

Self‐efficacy is a strong predictor of the level of accomplishment that individuals attain (Bandura et al., 1999). Nurses may fail to apply their knowledge and skills successfully unless they have an adequate level of self‐efficacy in tasks related to management of procedural sedation. The psychometric properties of the NC‐MSCS appear encouraging. Further testing of the instrument is required in different samples to provide further evidence of validity and to determine the minimal important difference. A particularly important further test of the validity of the NC‐MSCS will be to determine if higher levels of confidence (as assessed by the scale) are associated with safer patient care. This scale could be used to guide and inform education and training of nurses as well as complement formal competency assessment.

### 6.1 Relevance to clinical practice

The NC‐MSCS does not attempt to measure over‐complicated activities or tasks that go beyond nurses' knowledge or competences related to administering and monitoring procedural sedation, which have been outlined in clinical practice guideline statements from professional societies (Apfelbaum et al., 2018; Conway et al., 2013b). The 18‐item NC‐MSCS offers a very quick and simple tool for measuring self‐efficacy in performing such aspects of procedural sedation management. Although no prior research has specifically examined the effect of low levels of confidence towards the optimal management of sedation‐related complications, research from other similar clinical contexts provides some insight. For example, research in the resuscitation context has consistently demonstrated that lack of confidence is a barrier to achieving good quality cardiopulmonary resuscitation (Roh et al., 2012; Vaillancourt et al., 2013). Therefore, the NC‐MSCS could be a useful tool for self‐reflection, or to guide and inform sedation training initiatives and to inform formal competency assessment activities. An online version of the complete scale can be accessed and scored here. Also of note, considerable time and effort is directed in many hospitals towards annual competency assessments for a variety of aspects of clinical care. Competency in procedural sedation is one such area. The timeframe for such assessments, however, seems to be arbitrarily set and there is limited consideration of tailoring assessment to nurses individual capabilities and education needs. Application of the NC‐MSCS in education practice could be useful as a tool to stratify more experienced nurses to receive either further education and training in sedation (low confidence) or straight to simulation testing to ensure inaccurate calibration (high confidence but poor performance) is not an issue. Further research would be required to evaluate the efficacy of this approach.

## CONFLICTS OF INTEREST

The authors declare no competing interests.

## AUTHOR CONTRIBUTION

AC: Study design, data analysis and wrote manuscript. KC: Revised the manuscript for important intellectual content. NK: Revised the manuscript for important intellectual content. JS: Study design, data collection, revised the manuscript for important intellectual content.

## Data Availability

Availability of data and material (data transparency): All data used is available at https://github.com/awconway/nc‐mscs. Code availability: Code to reproduce the analyses is available at https://github.com/awconway/nc‐mscs.
